# Improving Suicide Prevention in Primary Care for Differing Levels of Behavioral Health Integration: A Review

**DOI:** 10.3389/fmed.2022.892205

**Published:** 2022-05-27

**Authors:** Margaret Spottswood, Christopher T. Lim, Dimitry Davydow, Hsiang Huang

**Affiliations:** ^1^Department of Psychiatry, University of Vermont Medical Center, University of Vermont, Burlington, VT, United States; ^2^Department of Psychiatry, Community Health Centers of Burlington, Burlington, VT, United States; ^3^Department of Psychiatry, Cambridge Health Alliance, Harvard Medical School, Cambridge, MA, United States; ^4^Executive Leadership, Comprehensive Life Resources, Tacoma, WA, United States

**Keywords:** suicide prevention, primary care, behavioral health integration, collaborative care, population health

## Abstract

**Importance:**

Suicide prevention implementation in primary care is needed due to the increasing rate of suicide in the past few decades, particularly for young and marginalized people. Primary care is the most likely point of contact for suicidal patients in the healthcare system. Attention to the level of medical integration with behavioral health is vital to suicide prevention and is applied throughout this review.

**Methods:**

A narrative review was performed.

**Observations:**

Many interventions help improve suicide prevention care. PCP education, screening, safety planning/lethal means reduction, care transitions, psychotherapy, and medication management are all evidence-based strategies. Additionally, the pragmatic topics of financing suicide prevention, supporting providers, enacting suicide postvention, and preparing for future directions in the field at each level of primary care/behavioral health integration are discussed.

**Conclusions and Relevance:**

The findings are clinically relevant for practices interested in implementing evidence-based suicide prevention strategies by attending to the behavioral health/medical interface. Leveraging the patient/provider relationship to allow for optimal suicide prevention care requires clinics to structure provider time to allow for emotionally present care. Defining clear roles for staff and giving attention to provider well being are also critical factors to supporting primary care-based suicide prevention efforts.

## Introduction

There is a pressing need for implementing evidence-based suicide prevention in primary care. There has been a 36.7% increase in the suicide rate within the past 20 years in the United States (1, 2). Suicide is the second leading cause of death in people ages 15–34 years and the tenth most common cause of death overall (3). Primary care is the most likely point of contact for suicidal patients in the healthcare system, with 77% of patients who die by suicide presenting to primary care in the year prior, and 45% presenting within the month prior to death (4). While the suicide prevention literature available is extensive, there remain few articles focused on the implementation of suicide prevention care in primary care systems that have varying degrees of behavioral health integration.

Compliance standards for suicide prevention mandates are increasing in healthcare (5). Fortunately, effective scalable practice interventions exist, and newer interventions show promise to improve suicide prevention (6). When one healthcare system mobilized multiple systemwide interventions with zero suicides as a goal, this rate was achieved and sustained for years (7). Suicide prevention strategies become easier to implement when primary care practices have the ability to track patients and collaborate with behavioral health providers.

To provide suicide preventive care in a primary care setting, some type of relationship with behavioral health specialists is critical. The suicide prevention literature thus far has not directly addressed how to provide preventive care considering the varying levels of behavioral health integration in practice. The relationships between primary care teams and behavioral health colleagues like psychologists, social workers, and psychiatric providers exist on a spectrum of informal to fully integrated. This article organizes achievable evidence-based interventions for primary care practices based on their current level of behavioral health integration.

There are six levels of identified mental health/primary care integration identified by the SAMHSA-HRSA Center for Integrated Health Solutions and summarized with permission in Table 1 (8). For the purposes of this article, we address three different levels of organization between primary care/mental health systems: (1) detached but with some coordination (“Informal Coordination”) (2) co-located (“Co-Location”) and (3) fully integrated (“Integration”).

**Table 1:** Levels of integrated healthcare framework.

	**Informal: minimal integration some distant coordination**	**Co-located: basic integration** **On site coordination**	**Integrated: formalized integration** **Fully merged collaboration, transformed practice**
BH/PCP/Provider Work	Separate facilities Rare to occasional meetings	Same facility Need drives communication Some shared administrative resources Informal interactions to help care for patients	Share space Joint solutions Function as one integrated system Regular team meetings and communication Shared concept of team care drives collaboration Blended roles
Clinical Delivery	Separate screening Formal requests to share information Separate care responsibilities Some shared knowledge for high utilizer patients	Agree on some screenings/criteria for in-house referral Some collaborative treatment planning for some patients Some focus on evidence-based population needs training	Consistent cross-discipline screening guides interventions Joint monitoring of target health conditions Standard population medical/behavioral health screening Consistent protocols One treatment plan Team selected evidence-based practices
Practice/ Organization	No coordination, collaborative onus on each provider Practice leadership might work toward systematic information sharing/valuing access to needed information	Co-location viewed as a separate project Leaders may be supportive of mutual problem solving of system barriers Inconsistent provider buy-in	Organizational leaders have strong support for integrated practice All providers engaged Strategy change: provides service delivery change until all providers embrace care components
Business Model	Separate funding and billing Specific project resources or facility expenses may be shared	Separate funding and billing May move toward sharing infrastructure costs	Blended/Integrated funding based on multiple sources Whole practice resource sharing Billing maximized for integrated model

*Adapted with permission from SAMHSA-HRSA 2020 (8)*.

The purpose of this article is to present and synthesize a comprehensive review of the literature on suicide prevention in primary care with different recommendations depending on the level of behavioral health integration. Many countries continue to operate behavioral health and medical services in a non-integrated fashion. In the United States today, with its fragmented healthcare reimbursement system, all three different levels of organization exist (Informal Coordination, Co-Location, and Integration). Efficient implementation for evidence-based interventions is recommended for these three levels of primary care/behavioral health collaboration. Financing of suicide prevention in the United States is discussed, as poor understanding of reimbursement is often an implementation barrier. Support for primary care providers asked to take a more active role in suicide prevention is presented as key to workforce sustainability. Finally, future directions for suicide prevention are discussed, focusing on how practices might prepare now for ensuing innovations. This paper should prove useful to clinic administrators such as medical directors and practice managers, primary care providers, and those involved in shaping suicide prevention policy. Additionally, suicide prevention resources are made available throughout the paper and in Appendices 1, 2.

## Methods

A narrative review of the literature was conducted due to the broad scope of the topic. The search strategy involved identifying previously published clinical guidelines, systematic reviews, studies of novel primary care-based interventions, and other primary literature to answer the question of how to implement interventions to decrease the risk of suicide for primary care patients.

PubMed and Google Scholar were searched, focusing on seminal articles in the past 20 years with particular attention to recent evidence-based improvements in care. Searches included combinations of the following terms and variations thereof: “suicide,” “primary care,” “prevention,” “screening,” “technology.” Bibliographies of relevant articles were also reviewed to identify additional appropriate references and primary literature.

Studies were reviewed by authors MS, CTL, and HH, taking into consideration strengths and limitations of study designs, relevance to improving care provision and readiness for new innovations in the primary care setting. Articles were excluded if they did not suggest interventions feasible in a primary care population. Articles were included if they described evidence-based screening and suicide prevention interventions or added depth to understanding an important topic.

## Summary of Findings

### Universal Standards for Suicide Prevention in Primary Care

While some interventions will be more feasible to implement in practices with higher levels of integration, universal evidence-based standards are recommended for all practices. Standards include primary care provider (PCP) education and practice-wide implementation of screening and effective interventions to reduce risk. Risk reduction interventions include safety planning with removal of lethal means and providing caring contacts around higher-risk care transitions (e.g., following a psychiatric inpatient or emergency room discharge) (6). Evidence-based therapy, medications, and suggested financing for suicide prevention are also offered in this section.

### PCP Education

In a recent meta-analysis, general practitioner and non-psychiatrist physician education on suicide prevention was found to be an effective suicide prevention intervention (6). In one older study, suicide was explored by primary care providers with only 36% of depressed patients (10). Physicians were more likely to inquire about suicide if they had had personal experience with depression, if the patient made a request for an antidepressant, or if the patient met criteria for major depressive disorder (10). Suicide care improves when systems improve, and one of the first steps is PCP education in suicide identification and management (11–13). Once PCPs feel sufficiently trained, they have increased clarity, job confidence, and positive attitudes about their efficacy in preventing suicide (14). Physicians receive effective one-time training in many topics, but standalone one-time training has been demonstrated to be insufficient for suicide prevention (9, 12, 15–18). Repeating educational sessions over years continued to correlate with a decrease in suicide (6, 9, 19). Training individual providers is an essential element in suicide prevention and educational efforts must be sustained to ensure continued impact.

PCP training should include the topics of screening and treatment of depression and substance use disorder (SUD), with psychiatrists available to supplement this treatment for more complex patients (6) (see Box 1).

Box 1 **PCP education based on level of collaboration**

**Table:** 

**Level**	**Implementation of PCP education**
Informal coordination	Recurring CME opportunities within or outside of the practice to learn about depression screening and treatment, substance use disorder (SUD) screening and treatment, and suicide prevention; annual PCP training may be sufficient to sustain change in practices (6, 9).
Co-location	Meetings 1+ times yearly with PCP/behavioral health together to learn about depression screening and treatment, SUD screening and treatment, and other suicide prevention strategies.
Integration	Ongoing collaborative depression and SUD screening and treatment with patient tracking, ongoing team-based suicide prevention teaching/learning, regularly scheduled review of high-risk populations with treatment changes recommended if patients are not responding. Ongoing quality improvement initiatives targeting at-risk patients. Yearly CME sessions to refresh the team's suicide prevention skills.

## Screening

### Whom to Screen?

The United States Preventive Service Taskforce (USPSTF) recommends universal depression screening for adults in primary care when paired with resources for diagnostic accuracy, treatment, and follow-up (20). Universal depression screening in primary care for adolescents ages 12+ is also recommended by the USPSTF and endorsed by the American Academy of Pediatrics (21). Data suggest that depression screening and treatment prevents suicide (6).

In 2014, the USPSTF found insufficient evidence to recommend for or against *universal* suicide screening in primary care for adolescents, adults, and older adults who lack a mental health diagnosis (22). A 10-year systematic review on suicide prevention strategies (23) and a review of U.S. Veteran primary care populations (24) resulted in the same conclusion. The case for *universal* suicide screening remains controversial within the suicide prevention community since suicidality can occur in the absence of identifiable risk factors (25, 26). One study found that only 39.5% of medically hospitalized adolescents (ages 10–21) who screened positive for suicidality also met criteria for depression (27).

However, screening for *high-risk groups* is agreed upon as the standard of care. *High-risk groups* in this context are those with any mental health or substance use disorder or taking a psychotropic medication (12, 28–31). In one cross-sectional study of 74 German primary care practices, additional predictors of suicidality included depression severity, male sex, and physical pain (32). More recent population analyses indicate rates increasing at a higher rate in Black and Asian or Pacific Islander youth, particularly female youth (33). Other recent population level data show that rates per sub-population vary significantly by geographic location (34).

The Perfect Depression Care Initiative in the Henry Ford Health System screens populations at risk, then stratifies patients into acute, high, moderate, and low risk for suicide based on screening, risk factors, and protective factors (7). Thereafter, the program immediately connects patients with targeted care based on these risk levels (35). Clinics should make sure patients who belong to populations with risk factors are screened.

### What Suicide Screening Instrument(s) to Use?

Many evidence-based suicide self-report screeners are available to identify suicide risk and fulfill the current standard of care (36); clinics can take into account their patient population and provider workflow when selecting a screening instrument (37).

#### PHQ-9 With Optional Suicide Screening Augmentation

Because the PHQ-9 is already used in many health care systems for depression screening and contains question #9 (“thoughts that you would be better off dead, or thoughts of hurting yourself in some way”), many studies use question #9 to detect suicidal thoughts (38–41). When question 9 was examined in an observational analysis of data from a patient registry of a collaborative care program for safety-net primary care patients (*n* = 11,015), suicidal thoughts were present in 45.2% of patients on initial assessment (39). Of those with a positive question 9, 5.4% did not meet screening criteria for depression, and only 2.2% of people who indicated the highest score for question 9 would have been missed through PHQ-2 screening. Question 9 of this instrument has demonstrated effectiveness in predicting suicidal behavior across minority racial and ethnic groups (42).

While Question 9 of the PHQ-9 is a convenient suicide screening question, it is not precise, asking about two variables at once (self-harm and wanting to be dead), and does not directly assess whether a patient wants to kill themselves (27, 28, 38, 39, 43–47). Despite these limitations, because of the ubiquity of the PHQ-9 for depression screening and treatment response measurement, using question 9 as a suicide screening question with additional follow-up screening available for a positive screen is a pragmatic approach.

Suicide researchers examined the administrative burden of asking all hospitalized medical patients about suicide directly (48). Adding four suicide screening questions (to the PHQ-9) administered by nurses in a hospital setting took 2 min for a negative screening and 4 min for a positive screen with no patients in the study requiring acute intervention (48).

#### P4

Created for use in primary care and specialty medical populations, this questionnaire is triggered when patients answer yes to having thoughts of hurting themselves (49). It helps the clinician evaluate past suicide attempts, suicide plan, probability of completing suicide, and preventive factors. It then stratifies risk into three categories: minimal, lower, and higher. In a study 0.4–1.6% of patients screened met criteria for higher risk, triggering emergency intervention (49). Preventive factors for those with high scores included faith, family, hope for the future, and fear of attempt failure (49).

#### Ask Suicide Screening Questions (ASQ)

The ASQ has been approved by the Joint Commission for youth and adults and has been studied in inpatient medical settings for adults (2-item version) (48) and outpatient settings for youth ages 10–24 (4-item screening version) (50). It has high sensitivity and negative predictive value (50–52).

Following a positive screen, the ASQ toolkit helps the team conduct a brief suicide safety assessment (BSSA) which determines next steps (acute positive screen with immediate intensive intervention or non-acute positive screen with brief suicide safety assessment to determine if full mental health assessment is needed), as well as providing all patients with a suicide prevention resource list (53).

In a recent retrospective analysis of patients aged 10–17 years given the ASQ (*N* = 91,580) across a safety-net public academic healthcare system, an emergency department, and 20 community outpatient clinics, universal screening resulted in 3% of patients being at any risk of suicide and acute positive screens in 1% of patients encounters (54). One in four patient encounters with a presenting psychiatric complaint resulted in a positive screen while only 2.7% of other encounters did.

#### Columbia-Suicide Severity Rating Scale (C-SSRS) Screen Version

Another suicide screening tool with a broad evidence base is the C-SSRS, which is validated for patients ages 12+ (55). It has been used extensively in research studies and can also be used by lay people, in healthcare settings, and in other community settings. A screening version is available that comprises two stem questions with four follow-up questions if the stem questions are positive, and one follow-up question if the stem question is negative. The triage points for primary care suggest three paths: (1) no intervention/referral needed, (2) non-urgent behavioral health referral or (3) urgent behavioral health consultation and patient safety precautions.

### Screening and Treatment for Alcohol Use

One qualitative study noted that heavy episodic drinking often occurred prior to unplanned suicide attempts (56). Alcohol use is not consistently assessed in primary care, and far fewer patients receive the recommended care for alcohol use disorder than for medical disorders (12, 57, 58).

Twenty percent of primary care patients have been found to drink in excess of National Institute on Alcohol Abuse and Alcoholism guidelines (59), resulting in the USPSTF recommendation that some level of screening for alcohol use be performed for each primary care patient >18 years old (60, 61). While a full discussion of screening and treatment for alcohol use is outside the scope of this narrative review, our recommendation includes using the 10-question Alcohol Use Disorders Identification Test (AUDIT) screen to further quantify alcohol use (60). Patients are provided increasing levels of support based on alcohol use risk scores (62). Recommended treatments include motivational interviewing and, for those with alcohol use disorder, behavioral treatment, support groups, pharmacotherapy (naltrexone, acamprosate, or disulfiram) and detoxification if needed (63).

### Screening for Firearm Access

Many primary care patients who subsequently die by firearm suicide will honestly answer screening questions about firearm access (64). Because of the high fatality linked to firearm ownership, recent evidence suggests that screening all primary care patients for firearms ownership with appropriate follow up care may prevent death from suicide for those who do not have a mental health or SUD diagnosis.

### Suicide Assessment Five-Step Evaluation and Triage (SAFE-T)

This is a pocket guide to help clinicians more thoroughly assess a higher risk patient to (1) identify risk factors; (2) identify protective factors; (3) conduct suicide inquiry; (4) determine risk level/intervention, and (5) perform documentation (65, 66). This resource aids behavioral health clinicians and medical providers in performing safety assessments.

#### Screening Summary

In summary, the best way to screen for suicidality by self-report continues to be an active research question and is dependent on the particular risk/benefit ratio based on specific primary care populations and how immediately available the behavioral health resources are in a specific primary care clinic. Universal depression screening and treatment is the standard of care and helps prevent suicide. Patients presenting with a mental health concern, with a mental health diagnosis, with a substance use disorder, or prescribed a psychotropic medication should be screened specifically for suicide risk in primary care. If the primary care population is a higher-risk population, it is recommended that 2–4 suicide screening questions are automatically administered following the PHQ (see Box 2).

Box 2 **Suicide risk screening based on level of collaboration**

**Table:** 

**Level**	**Implementation of suicide prevention screening**
Informal coordination	Universal screening and treatment of depression using screening with a built- in triage component for active suicidal ideation for those at elevated risk based on PHQ-9 question #9 or other high-risk factors (e.g., active substance use). Use a meeting or educational session to “dry run” protocols for different screening outcomes to connect patients with indicated services.
Co-location	Universal screening and treatment of depression, with added suicide screening for those at elevated risk. Consider coming to an agreement with the co-located mental health service providers regarding timely next steps for care for high-risk patients and practicing warm hand-offs. Practice protocols for what to do when patients screen high risk for suicide.
Integration	Universal screening and treatment of depression and added suicide screening for those at elevated risk. Workflow includes regularly reviewing a list of higher-risk patients based on screening outcomes and proactively tracking these patients to change treatment if patients are not responding to current interventions. Practice protocols for what to do when patients screen high risk for suicide.

## Risk Reduction Interventions

Patients will be stratified by risk after screening. Those with acute risk should be transferred to a higher level of care such as a behavioral health specialist safety assessment and/or emergency services. Patients with non-acute risk should complete a brief suicide safety assessment to determine if, and how soon, a comprehensive mental health evaluation is needed (67). Interventions for non-imminent risk that can be provided in the primary care setting include safety planning, removing lethal means, and timely contacts targeting care transitions (36). Genuine emotional connection with clinic staff and providers is important throughout this process and is discussed further in the “support for providers” section.

### Safety Planning Intervention (SPI)

After a patient is identified as being at elevated risk for suicide, immediate safety planning during the visit is recommended. When separate from ongoing psychotherapy, such safety planning is called a Safety Planning Intervention (SPI) (68). This consists of a written list of coping strategies and sources of support that are prioritized to help patients address a suicidal crisis; this brief intervention should be done at the PCP office as suicidal patients do not often follow up with behavioral health referrals (68–71).

Safety planning should not be confused with a “contract for safety/no-suicide contract,” which has been shown to be ineffective (73). Safety planning requires that practice managers designate a work-flow to accomplish this when needed, and allow for building in mental health support staff based on local resources (68); practices without behavioral health support should determine in advance who will create the safety plan with the patient (e.g., nursing staff, trained medical assistants, PCPs). The practice can review standardized forms like the safety plan script and resource list from the ASQ Toolkit (53) and the Brown-Stanley Safety Plan Template (71). Safety planning includes helping the patient write down warning signs, internal coping strategies like writing and self-soothing, external coping like distraction via people and social settings, individuals to call for help, professional resources to contact, and environmental lethal means reduction. Families and other social supports are important participants in safety planning, with the patient's permission. The Perfect Depression Care Initiative in the Henry Ford Health System provides a handout called “Understanding and Helping Someone Who is Suicidal” which includes information about warning signs and feelings, action steps, how to work with the treatment team, and whom to contact if a person feels suicidal (7). Suicide prevention literacy of providers, patients, and social supports drive success in brief contact interventions (74). Primary care practices can put in place protocols to enlist families and other social supports if the patient grants permission.

### Remove Lethal Means

This is part of safety planning but addressed separately in this review as the nuances are such that primary care clinics may consider additional training for staff. Primary care clinics can endorse public health interventions like pharmacy/police collection boxes for extra medications and firearms as safety measures. Suicide attempts often occur during an acute crisis; 52% of completed suicides occur via firearms, 23% occur via suffocation, 18% via poisoning/overdose, 2% via jumps, 2% via cuts, and 4% via other means (75). Ninety percent of those who survive a first attempt do not subsequently die by suicide (76). When public health interventions target decreasing access to lethal means, fewer people complete suicide (77–80).

Broaching how to decrease access to lethal means with a patient is a specific skill set for integrated behavioral health clinicians or for medical clinicians and staff in practices without on-site behavioral health support. The Counseling on Access to Lethal Means (CALM) 2-h training course was created to provide this skill set. CALM focuses on the evidence behind reducing access to lethal means to prevent suicide, practicing strategies for conversations, off-site and in-home storage options for firearms and dangerous medications, and plan with patients and families to increase follow up and reduce access to lethal means (81).

### Care Transitions

The USPSTF has called for research focusing on behavioral health care transitions (22, 37). Transitions of care between the psychiatric inpatient unit or emergency room and outpatient care are times of increased risk for suicide (82). The above interventions as well as caring contacts (see below), timely follow-up visits, and contacting the patient if they miss a visit should be employed during these times of higher risk. Primary care offices should plan for “wraparound care” of those identified as at acute risk. If a care manager is part of the team, this can include frequent check-ins with the patient and coordination between any systems serving the patient (e.g., inpatient hospital, therapist, or crisis team) (see Box 3).

Box 3 **Safety planning interventions and lethal means reduction based on level of collaboration**

**Table:** 

**Level**	**Implementation of safety planning and lethal means reduction**
Informal coordination	PCPs and nursing staff become adept at creating safety plans with patients who screen as at risk but not at imminent risk (72). PCPs and nursing receive training such as CALM on counseling to reduce access to lethal means and practice these conversations.
Co-location	Consider if behavioral health colleagues will regularly train co-located PCPs and nursing regarding safety planning and lethal means reduction; consider CALM training for both PCPs/nursing staff and behavioral health colleagues.
Integration	Behavioral health staff are available for safety planning and lethal means reduction for patients when needed. PCPs and nursing should also be knowledgeable about how to do this if behavioral health staff are not available. Consider CALM training for all patient-facing clinical staff (medical and behavioral health).

### Caring Contacts

Once patients are identified as being at increased risk for suicide, reaching out at several specific times to provide brief, non-demanding communications voicing concern and care have shown to improve outcomes (83–88). This has been studied using postcards, telephone calls, and text messages. Systematic ways of implementing caring contacts can remove the burden of care from providers alone (83). Helping patients remember that they matter to providers leverages the patient-provider relationship against suicide. There are free examples of text for caring contacts available for clinics to implement this intervention (e.g., Caring Contacts Text and Scripts are available at nowmattersnow.org) (see Box 4).

Box 4 **Care transitions and caring contacts based on level of collaboration**

**Table:** 

**Level**	**Implementation of care transitions and caring contacts**
Informal coordination	Primary care practice makes sure care transition documents from the hospital are available. PCP has a follow-up appointment soon after a psychiatric emergency visit or psychiatric hospitalization. Using a list of high-risk patients, primary care practices encourage and track patient engagement in scheduled behavioral health appointments and send caring contact postcards or place follow-up phone calls at regular intervals for the next year.
Co-location	See suggestions above. If a patient has follow-up appointments with co-located behavioral health providers, PCP discusses the patient's needs with behavioral health providers and negotiates who will send the caring contacts based on the strength of the relationship with the patient.
Integration	Transition documents from the hospital are available to the team. The patient is placed as higher priority on the care registry to review frequently in collaborative meetings and make sure follow-up care is effective. Caring contacts are sent from a team member with whom the patient has a good relationship (e.g., care coordinator, PCP, or integrated therapist) at intervals for the following year.

### Psychotherapy

The Zero Suicide model recommends that primary care clinics without integrated behavioral health services establish relationships with local crisis lines, therapists, and other behavioral health partners. Therapies with evidence to help prevent suicide include cognitive therapy-suicide prevention (a form of cognitive behavioral therapy, or CBT) (6, 69, 89), dialectical behavior therapy (DBT) (6, 90), problem solving therapy (91), mentalization based treatment (92), and psychodynamic interpersonal therapy (93, 94). CBT and DBT are recommended as the most scalable options (6).

Psychotherapy, a relatively resource-intensive treatment, should be recommended to those with high suicide risk who do not currently require a higher level of behavioral health care (such as an intensive outpatient program or inpatient hospitalization). In order to provide immediate therapy and medication management for those at risk, the Perfect Depression Care Initiative in the Henry Ford Health System offered at least one 90-min weekly drop-in group at each outpatient clinic run by a psychiatrist and social worker for immediate access to evidence-based therapy and medication management (7). One meta-analysis demonstrated that general group treatment decreased suicides in one study, decreased suicidal ideation in half of studies, and had no effect on non-fatal suicide attempts (6). Groups can be offered as an immediate scalable intervention, but more robust research is needed regarding which patients most benefit from this treatment and which will need additional interventions.

### Medications

Finding and treating the modifiable factors of suicidal ideation has been the standard of care, with particular attention given to depression care. Recent thinking around suicide prevention increasingly focuses on treating suicidal ideation in parallel to treating the presumed underlying disorder (95). Medications with the potential of lowering risk of suicide include lithium in patients with bipolar disorder (95, 96), ketamine in patients with severe major depression (95, 97), and clozapine in patients with schizophrenia (95, 98).

The combination of therapy and appropriate medication for identified psychiatric conditions has been demonstrated not to be superior to either alone for suicide prevention (6).

## Financing Suicide Prevention

The following section on the financing of suicide prevention is specific to the United States. Country-specific literature regarding how different payment structures drive behavioral health/medical integration are required when examining this question for other systems of care.

### Cost-Effective Primary Care Interventions

In the United States, safety planning and caring contact follow up phone calls are relatively low intensity and have been demonstrated to be cost effective in fee-for-service primary care settings if the suicide risk prediction used has a specificity of >94% and sensitivity >16%, with a PPV of 1% or greater (99). More intensive intervention risk reduction included CBT or DBT therapy, which, to be cost effective, requires the suicide risk prediction method to have a specificity of 95% and a sensitivity 35% or greater, resulting in a PPV of 2%. A PPV of 2% is similar to other primary prevention risk thresholds with rare but high-cost negative outcomes (99, 100).

### Billing

With the current fee-for-service reimbursement model in much of the United States, billing for suicide prevention is an important consideration. Comprehensive coding flow diagrams can be found for standalone primary care clinics and for integrated primary care/behavioral health clinics on the Zero Suicide website (101). See Table 2 for a summary of currently available codes (101) that are currently available (see Box 5).

**Table 2:** United States reimbursement codes for any behavioral health integrated level.

**Code**	**Use**
GO444 (15 min) G8431(HD modifier) G8510 (−screen, HD modifier) G8431 (+ screen, HD modifier)	Annual depression screen using PHQ-2
96127	Suicide risk high, further suicide screening with behavioral health assessment and documentation
99213-99214	PCP code for higher EM time/complexity when PHQ-9 question #9 is positive or other concern for suicide
90792 (full biopsychosocial assessment) 99205 (new patient visit, Medicare) 99214 or 99215 (EM code for time/complexity)	Psychiatric Provider visit
90839 (60 min for psychiatric crisis) 90840 (added complexity for crisis/time) OR 90847 (45-min psychotherapy code) 90791 (full biopsychosocial assessment)	Behavioral health provider (non-prescriber) visit
90834 BH Visit 90839/90840 Crisis visit	Complete suicidality scale (e.g., C-SSRS) since last visit, update risk assessment and safety plan
96127	Brief assessment for suicide risk (can be used 4x yearly) or other brief assessments of mental illness
90834 BH Visit 90839/90840 Crisis visit	Complete suicidality scale (e.g., C-SSRS) since last visit, update risk assessment and safety plan
96127	Brief assessment for suicide risk (can be used 4x yearly) or other brief assessments of mental illness
G8431 (with HD modifier)	Clinical depression screening is positive, with follow up plan
G8510 (with HD modifier, replacing 99420)	Clinical depression screening is negative
96127	Screening with brief emotional assessment including scoring/documentation on a standardized instrument
96191	Caregiver-focused health risk assessment instrument Maternal depression screening during well-child visit, billed under child
99452	Referring or treating provider spends 30+ min providing patient information to a consultant aided by relevant electronic media

*BH, Behavioral Health; C-SSR, Columbia-Suicide Severity Rating Scale; EM, evaluation and management provided by a physician or other qualified health professional; HD, pregnant/parenting women's program; PHQ, Patient Health Questionnaire*.

*From the Educational Development Center (EDC) Zero Suicide Institute. Financing Suicide Prevention. Available online at: https://zerosuicide.edc.org/resources/key-resources/financing-suicide-prevention*.

Box 5 **Psychotherapy and medication treatment based on level of collaboration**

**Table:** 

**Level**	**Psychotherapy and medication treatment**
Informal coordination	Send an introductory letter to potential behavioral health partners to establish a relationship. Establish relationships with local crisis lines and providers (see a sample behavioral health outreach letter from the Suicide Prevention Resource Center which is available in Appendix 2). Be familiar with first- and second-line medication management for depression and other common psychiatric disorders. Explore if there is a psychiatrist available to consult with for treatment-resistant moderate or severe psychiatric illness.
Co-location	Meet and/or send an introductory letter to co-located behavioral health partners. Be familiar with first- and second-line medication management for depression and other common psychiatric disorders. Explore if there is a psychiatrist available to consult with for treatment-resistant moderate or severe psychiatric illness. Patients with increased psychiatric illness severity may require a higher level of psychiatric care (e.g., through local behavioral health agencies or tertiary medical centers). Co-located behavioral health practitioners may consider CBT/DBT/medication management drop-in groups weekly with rolling admission for rapid access when needed while awaiting individual treatment.
Integration	Establish relationships with local crisis lines and providers. Embedded therapists provide evidence-based short-term therapy for depression and other common behavioral health disorders. Track patients on psychiatric medications weekly and make an evidence-based change if patients are not improving. The care coordinator reviews patients with a consultant psychiatrist regularly. Patients with increased psychiatric illness severity will likely need a higher level of psychiatric care with local behavioral health agencies or university programs. Run CBT/DBT/medication management drop-in groups for immediate access while awaiting individual treatment.

### Financing the Collaborative Care Model

Primary care clinics that have implemented the Collaborative Care Model (CoCM) may have higher capacity to provide and follow up on rapid, targeted treatment for those with suicidal ideation and/or depression symptoms (12, 25, 29, 30, 39, 72, 102). Collaborative care is an integrated care model in which a team consisting of primary care providers, care coordinators/case managers, and psychiatric consultants work together providing screening, regular/proactive monitoring and treatment, and psychiatric caseload reviews, to target care to outcomes with evidence-based techniques (103). This has been shown to improve mental health outcomes (103).

Practices adopting CoCM still require the ability to refer the highest-risk and highest-complexity patients to community behavioral health providers for appropriate ongoing specialty services. CoCM does not address all behavioral health needs in a population but is designed to provide effective care in the primary care setting, thus enabling specialty behavioral health resources to be devoted to the highest-severity patients.

Reimbursement for team-based care and population health management programs is progressing at different rates across the United States. Accountable Care Organizations (ACOs) are one such model that incentivizes primary care clinics to take on population health management (104). To fully adopt these models, primary care-based data systems to measure per member per month utilization and cost are needed (104). However, there are billing codes for team-based population behavioral health care within the current fee-for-service system. New codes were introduced in 2017 and, in some U.S. states, integrated primary care/behavioral health can be reimbursed for work undertaken together (105–107).

Different types of practices may have different code availability, not all payers or states reimburse for each code, and the AIMS center and the CMS Medical Learning Network will provide the most up-to-date information as these codes evolve, see Table 3 (106, 107) (see Box 6).

**Table 3:** United States integrated team-based reimbursement codes.

**Code**	**Use**	**Behavioral health care manager/clinical staff time**	**Assumed billing practitioner time**
99492	First CoCM month	70 min per month	30 min
99493	Subsequent CoCM months	60 min per month	26 min
99494	Add-on CoCM any month for extra time	Each additional 30 min per month	13 min
G2214	Initial or subsequent collaborative care	30 min per month	Usual work for the visit code
99484	General behavioral health integration	20+ min per month	15 min
G0511	General care management services for FQHC practices	20+ min per month	Usual work for the visit code
G0512	Psychiatric CoCM for FQHC practices	Minimum 70 min initial month and 60 min subsequent months	Usual work for the visit code

*Care Team, treating (billing); PCP, Behavioral Health Care Manager; Psychiatric Consultant, Beneficiary (patient)*.

*From the AIMS Center. Billing and Financing Behavioral Health Integration and Collaborative Care Available online at: https://aims.uw.edu/resources/billing-financing and from the Centers for Medicaid and Medicare Services MLN 909432 Available online at: https://www.cms.gov/Outreach-and-Education/Medicare-Learning-Network-MLN/MLNProducts/Downloads/BehavioralHealthIntegration.pdf*

Box 6 **Financing suicide prevention care based on level of collaboration**

**Table:** 

**Level**	**Financing Suicide Prevention Care**
Informal coordination	Regular education and monitoring for the billing department and clinicians around using appropriate PCP codes when screening for suicide and working with suicidal patients.
Co-location	Regular education and monitoring for the billing department and clinicians around using appropriate PCP and PCP/behavioral health codes when screening for suicide and working with suicidal patients.
Integration	Use PCP, PCP/behavioral health codes, and team-based care (CoCM) codes as appropriate. As relevant and feasible, work with payers, policymakers, and other state agencies toward coverage of team-based care models.

### Support for Providers Improves Engagement

Physicians are at higher risk of suicide than the general population and female physicians are at higher risk relative to males (108–110). Poor medical health, depression, and work stressors demonstrate a stronger association with physician suicide than with non-physician suicide (110). In one academic medical center, a majority of physician respondents to a wellness survey met criteria for being at moderate or high risk of suicide or depression, and though only 15% of these at-risk respondents engaged in mental healthcare offered; those who did were satisfied with the care (111). Workplace cultures that include a healthy environment and increased mental health support for workers may be particularly beneficial to clinicians and staff who do not regularly engage in self-care.

Increasing support for PCPs enhances clinician-patient communication. Relationships with providers who are caring and listen help patients disclose suicidality; data indicate that patients believe it is appropriate for providers to ask about suicidality, report that provider overreaction is a deterrent from further disclosure, and note that, when disclosures occur, they want to be helped but are worried about negative consequences (56, 112). Although PCPs are increasingly called upon to provide mental health care, they typically do not have time built into their clinic to receive supervision and support to reflect on the emotional interactions they have with patients (113). In a qualitative study patients noted that, when the provider asked about suicide risk “like reading off a script and… checking boxes rather than I'm afraid for your safety,” it seemed that the provider was only asking out of obligation (56). Conversely, when providers expressed genuine listening and caring, it was easier to be honest about their experiences, and “expressions of listening and caring were more important than finding an immediate solution to their problems” (56).

One way to help clinicians convey the listening, empathy, and respect needed to promote communication and increase hope for individuals contemplating suicide is for clinicians themselves to feel heard, have empathy from, and feel respect within their workplaces. Workplace cultures that acknowledge the reality of healthcare being complex and support knowledge improvement and emotional healing for patients, families, staff, and communities at large may lead to improved overall wellbeing and improved provision of effective suicide care (114). Restorative Just Culture has been used in one public mental health system along with a Zero Suicide framework. This model shifts the focus of a post-suicide inquiry from *who missed an opportunity to prevent the loss of life* to *what was responsible for a missed opportunity* by asking *who is hurt, what do they need*, and *who is responsible for meeting that need* (114). Overall, work is a major social determinant of health, and public health literature suggests that work redesign can address conditions that lead to stress and burnout in general (115). While a comprehensive discussion on addressing physician burnout is outside the scope of this paper, several important approaches include training and employer support to improve social relations at work, increasing worker schedule control and voice, and adjusting job demands (115). This theme of having systems responsive to the emotional health of workers overlaps with inclusion of a Restorative Just Culture alongside suicide prevention efforts, a concept discussed in the next section.

One qualitative thematic analysis of provider perspectives regarding factors important to suicide risk assessment and management highlighted the importance of team-based care, patient-provider relationships built on trust, integrated behavioral health providers giving education to PCPs about suicide prevention, the ability for patients to access mental health care, and system-wide prevention efforts (116). In addition to improved medical and mental health care and outcomes for patients, one of the secondary benefits of integrated team-based models is that consultation about patient care through regular case review also appears to be associated with favorable PCP experience (117).

Just as suicide prevention improves with system culture change (7, 72, 114), provider well-being and resulting ability to participate in mental health work can be best addressed at a clinical systems level rather than an individual provider level (95, 115) (see Box 7).

Box 7 **Support for provider wellbeing to improve clinician-patient communication based on level of collaboration**

**Table:** 

**Level**	**Support for providers to improve clinician-patient communication**
Informal coordination	Ability to pause clinic in the rare instance a provider needs extra time to connect emotionally with an acutely suicidal patient. Mechanism for behavioral health consultation and care transition.
Co-location	See above. Ability to consult co-located behavioral health providers regarding patients requiring a higher level of behavioral health care.
Integration	See above. Provider feedback to improve systems of care functionality. Case review used as a model of specialist consultation.

### Suicide Postvention

The Zero Suicide framework is both motivational and aspirational. With appropriate and rigorous care, suicides can dramatically decrease in populations (72). Tragically, just as with caring for ill medical patients who sometimes die, not every suicide can be prevented (114). Patient suicide has a profound impact on clinicians and healthcare workers (118–120). When suicide does occur, it is traumatic to providers, and trauma may lead people to isolate from one another (121). Because of suicide contagion, thoughtful suicide postvention may also serve as an antidote to this isolation and as suicide prevention for providers and others in the community.

One retrospective study found that PCPs feel grief, guilt, and self-scrutiny following a patient's death by suicide (122). Clinical staff can feel that they failed the patient and worry about their own clinical skills and choice of career (114, 123, 124). Amidst emotional loss, clinicians may fear or experience legal retribution, and possible loss of licensure, income, and reputation (114, 124–126). Most providers report turning to peers and colleagues for support following a patient's suicide (122).

Turner notes that, in the aftermath of a suicide, healthcare organizations may focus solely on documentation of risk prediction or lack thereof and overlook factors that have an equal or larger impact on suicide prevention (114, 126, 127). Helpful factors to examine include therapeutic relationships and instilling a sense of hope for future patients struggling with suicidal urges (114, 128). Primary care clinic administrators can help providers identify and go through the multiple elements of a comprehensive suicide postvention, including addressing emotional, professional, legal, and administrative consequences (129).

One model of organizational support (114, 128) for providers and other second victims like family/friends in suicide postvention is the TRUST acronym: just Treatment, Respect, Understanding and compassion, Supportive care, and Transparency/opportunity to contribute (114, 130). Postvention toolkits are available to provide evidence-based practices; toolkits include Texas' postvention toolkit, the Suicide Prevention Resource Center-endorsed practices, and the LOSS program (Loving Outreach to Survivors of Suicide program) (131) (see Box 8).

Box 8 **Suicide postvention based on level of collaboration**

**Table:** 

**Level**	**Suicide postvention**
Informal coordination	Use the TRUST acronym in a postvention response. Choose and use a postvention toolkit.
Co-location	See above. Include formal postvention processing with medical and mental health treaters who were impacted.
Integration	See above. Care coordinators can help with outreach to community survivors.

### Future Directions

There are numerous future directions for suicide prevention in primary care. For the purposes of this paper, we focus on the following: addressing underserved populations, improved detection/prediction, technology-based interventions, and shortening timelines of suicide risk detection and intervention.

Increasing identification and treatment engagement of underserved populations at risk of suicide should be prioritized. There is a pressing need to acknowledge and mitigate the social inequalities that impede provision of evidence-based treatment for those unjustly suffering the effects of systemic poverty and/or bias (95, 132). Suicide is increasing in Black youth (133, 134), and there are higher rates of suicide attempts among LGB people (135). Social adversity and clinician bias due to ethnicity contribute to screening and treatment gaps for higher-risk populations (136). Clinics can work to hire staff and providers that represent the demographics of the patient population served and to decrease attitudinal barriers for all staff by making sure suicide prevention and other trainings include opportunities to expand clinician and staff self-awareness around bias and anti-biased actions.

More research is needed to predict acute suicide risk; to date, only 0.1% of studies of suicide completion among those at elevated risk of suicide in the past 50 years have looked at suicide outcomes within 30 days, a clinically relevant timeframe, whereas most studies have assessed suicide completion over longer timelines (137). Interactive digital tools may activate patients to increase discussion of suicidal thoughts with PCPs (138). Such programs might be offered in waiting rooms or through social media algorithms to help with identification. Due to the need to improve interventions prior to the first suicide attempt and the waxing and waning nature of suicidality, digital phenotyping via smartphones also holds promise as a future intervention (139, 140). There is also ongoing research to identify the most effective treatment for each patient among those at risk of suicide (140, 141).

While self-report is an important method to identify suicidal ideation [>60% of people who attempt suicide seek help first (142, 143)], subjective markers of suicidality have limitations. People lack conscious awareness of factors that influence their behaviors (144, 145), they may have motivation to deny or conceal suicidal thoughts [78% deny these in their last communication prior to death (144, 146)], and suicidal thoughts are transient (147). An implicit association test (IAT) asking patients to pair “me”/“not me” and “life”/“death” has been tested in a variety of settings as a behavioral measure of suicide prediction. Results demonstrate difficulty differentiating between patients who have suicidal thoughts and those who will attempt suicide (144). There may be clinical utility using incremental prediction (145, 148) for this test when paired with other methods of suicide prediction, but more research is needed.

Machine learning algorithms to predict suicide risk can take into consideration known risk factors, such as treatment history, family history, general psychopathology, prior suicidal thoughts and behaviors, social factors, physical illness, demographics, externalizing and internalizing emotional experiences, psychosis, and biological factors (137). Novel risk factors can also be identified (149). Evidence exists for machine learning algorithms in the EMR paired with the PHQ-9 question 9 resulting in effective screening for suicide in a college student population (38). Digital phenotyping may also contribute to machine learning and contribute to suicide risk prediction (141, 142).

Separate from EMR data mining, Computer Adaptive Testing for Mental Health (CAT-MH^TM^, Adaptive Testing Technologies, Chicago, IL, www.adaptivetestingtechnologies.com) asks patients to fill out a set of questions, the answers to which change subsequent questions in real time, allowing for a short and precise screen that can be repeated over time using different questions (150, 151). In one pilot RCT of 20 patients, CAT-MH demonstrated greater association with gold-standard diagnostic tools such as the PHQ-9 and C-SSRS (151). More research is needed prior to scaling this intervention, but the benefits of improved screening through an adaptive algorithm may enhance suicide risk identification. If subsequent studies continue to support this method, CAT-MH may help with rigorous, efficient screening, freeing up provider time for therapeutic interventions.

Overall, studies suggest that targeted provider-initiated screening of higher-risk primary care patients combined with universal EMR mining may be the most thorough and accurate approach to timely suicide risk identification in primary care (23). In essence, no single strategy is best alone, so practices would need the ability to combine evidence-based algorithms in the future (23).

Regarding future directions for treatment, the current standard of care includes targeted caring contacts (99) and safety planning, lethal means reduction, and appropriate referral (36). Technology may improve engagement in treatment for suicidal ideation. However, one randomized clinical trial found that EMR online messaging by care managers did not change suicide or self-harm risk while the online delivery of dialectical behavior therapy skills was associated with a significant increase in risk of severe self-harm (death or hospitalization) (152). Engagement with mental health treatment apps can be high (153–157) but, between 10 days to months after download, the level of engagement may decrease, resulting in retention rates of 50% or lower (158–160). Primary care practices should be discerning regarding how care is delivered and which suicide prevention and depression apps are recommended.

Most mental health and suicide monitoring apps have not been systematically vetted for safety and effectiveness (157, 161, 162). Furthermore, apps may focus on engaging individuals alone rather than on engaging friends, family and clinicians (157). Martinengo suggests that the level of app governance should match the potential risk/benefit to users and concludes that suicide prevention apps do not meet this standard and often do not include best practices for suicide prevention (163). However, there are several evidence-based apps containing many best-practice features (including means safety, support, crisis line access, treatment, and safety plan) (157). The Koko App targets increasing treatment engagement for those at risk (164) by using machine learning from social media posts to identify those “online and in crisis” but reluctant to engage in treatment (165). Use of the app resulted in a 23% increase in crisis service engagement.

As another opportunity for collaboration with specialty mental health providers, if supported by future research, inpatient psychiatric units may begin to collect real-time data about dynamic changes in suicidal ideation during hospitalization to characterize risk post-hospitalization (166). Primary care practices and/or community mental health providers could then provide more intensive care transitions for those at highest risk.

Future research should focus on identifying suicide risk within shorter timelines (e.g., within 30 days) and continue to consider more effective screening and risk reduction interventions for participants without technological literacy (141). Clinics should be ready for a layered approach to incorporating self-report data, electronic medical record (EMR) data harvesting/machine learning, and enhancing provider awareness through means such as the Implicit Suicidal Cognition Association Test (IAT) to make identification result in effective and timely intervention in the future (141). If a dynamic model (i.e., one that synthesizes data from multiple methods) turns out to be the most powerful suicide predictor, and interventions can be more targeted, primary care practices will need stronger data systems and population health capacities (see Box 9).

Box 9 **Clinical preparation for future directions**

**Table:** 

**Level**	**Preparation for future directions**
Informal coordination	Educate mental health agencies in the community regarding capacity to respond to improved monitoring. If upgrading an EMR, check for capacity to track patients and the ability to pivot toward emerging technologies.
Co-location	Discuss capacity to respond to improved monitoring of suicide risk, work together to move toward an EMR that can accommodate emerging suicide prevention technologies.
Integration	Monitor the state of the evidence around when to adopt new technologies, make sure the EMR allows for a team-based response when suicide risk is identified with suicide prevention technologies.

## Discussion

Current standards of suicide prevention rely heavily on effective coordination between primary care medical teams and behavioral health colleagues, making it essential that local levels of behavioral health integration (Table 1) are considered by practices during implementation efforts.

This review describes the evidence behind prevention strategies: PCP education, screening, safety planning/lethal means reduction, care transitions, psychotherapy, and medication management. We discuss how practices can finance suicide prevention, support providers, enact suicide postvention, and prepare for future directions in suicide prevention at each level of primary care/behavioral health integration. Based on this review, future directions in improving primary care-based suicide prevention include (1) implementation of evidence-based strategies in real-world settings, (2) increasing coordination between behavioral health and medical teams, and (3) prioritization of workforce (not simply individual) wellness to foster the genuine clinician-patient connection.

We identified that the existing literature either suggests all-encompassing clinic change (e.g., the Zero Suicide model) or individual provider-level change [e.g. (12)] but many lacked practical implementation strategies for real-world clinics. Recent literature shows that when social work is embedded into primary care practices, patients receive more rapid behavioral health treatment (167). Suicide prevention cannot wait for practices to adopt increasing levels of behavioral health integration, especially financial incentives are lacking. This review augments the existing literature by making implementation suggestions for practices based on level of current BH integration. The differences in training and implementation for practices with different levels of integration are vital. For example, with informal coordination, PCPs or adjacent staff will need the time and skills to complete the basics of suicide prevention (screening, safety assessment/lethal means reduction, care transitions and medication with access to rapid therapy referral). Alternatively, for fully integrated practices, embedded behavioral health providers may be able to take responsibility for care following a positive screen. With all levels of integration, seamless transitions between higher levels of care (i.e., emergency and inpatient) and outpatient primary care and behavioral health services should occur; however, roles may be performed by different staff, with a greater burden likely falling on PCPs for practices with lower behavioral health integration. Practices should place themselves on the spectrum of integration prior to implementing suicide prevention strategies to best assign and provide time for workload changes based on available staff. Future literature should focus more on the workings of the key relationships between PCPs, primary care practices, and behavioral health colleagues.

The literature indicates that greater integration of care between behavioral health and medical teams in real-world settings results in a range of benefits. Improving organization around care monitoring and delivery improves health outcomes. Without formalized integration, more suicide prevention work falls to PCPs. When feasible, we recommend integrated care and CoCM as part of population health management for depression (29) and suicide prevention (6, 12, 25, 30, 72, 102).

Greater integration of primary care and behavioral health care can enable efforts around primary care-based suicide prevention. On the one hand, some initiatives can be implemented regardless of the degree of integration, such as the provision of weekly drop-in DBT/CBT groups for at-risk patients identified in primary care, described in the Zero Suicide model (6, 90). By contrast, team-based approaches may facilitate clear designation of roles among the team, reducing PCP burden. Clinic models that track and respond to population health needs are more likely to have capacity to increase patient engagement, integrate new methods of screening (e.g., through application of machine learning algorithms to EMR data), and coordinate with behavioral health providers for monitoring of individuals at risk.

Finally, prioritizing workforce wellness to foster genuine clinician-patient connection is needed to support suicide prevention efforts. Without appropriate preparation and support, PCPs may under- or over-react to discussions about suicide. Suicide prevention requires staff and PCPs to have the emotional and temporal space to be present with patients. Providers and staff also deserve their own behavioral health support. Strategies to create healthy work environments have been identified in the public health literature (115) and overlap with those identified in the suicide prevention literature (114). Clinics can focus on changing the work environment to enable providers to have time for suicide prevention strategies, and for reflection to sustain this work over time. Future research should further explore the effect of healthy work environments on the connections with at-risk patients using evidence-based prevention strategies.

This paper focused on closing the gap in the current suicide prevention literature regarding behavioral health/medical interface, building on established knowledge. Limitations of this work include those of a narrative review including that the number of articles considered were not tracked and the strength/risk of bias of included articles were not evaluated. However, articles with strong methodologies including clinical guidelines, systematic reviews, and studies of novel primary care-based interventions were targeted for inclusion.

## Conclusions

Extensive literature exists on the effective primary care-based screening of suicide risk, intervention, support for PCPs, and future directions for primary care-based suicide prevention. Current challenges in the field include the need for improved identification and treatment of those at risk and suboptimal funding incentives for suicide prevention care. This review represents a novel contribution to the literature by describing the ways suicide prevention strategies can be implemented in the primary care setting at different levels of behavioral health care integration (informal coordination, co-location, and integration). Given the importance of genuine connection for suicide prevention, we suggest that principles of workplace wellness be adopted to prevent staff burnout as suicide prevention becomes part of everyday practice workflow. This review highlights the need for more literature on real-world implementation focusing on how PCPs and behavioral health providers interact and the need for policy and financial strategies to incentivize greater primary care/behavioral health integration. While primary care practices should not wait for broad policy changes to implement elements of suicide prevention, we recommend that work toward more integrated practices is aligned with suicide prevention improvement. Investing in suicide prevention is an investment in saving lives as well as the basic tenets of good care; strategies needed for suicide prevention help practices connect better with patients and become more responsive to provider needs.

## Author Contributions

MS researched, wrote, and edited the manuscript. CL researched and edited the manuscript. DD and HH provided conceptual guidance and edited the manuscript. HH provided additional support tracking references. All authors contributed to the article and approved the submitted version.

## Funding

This work was supported in part by the Four Pines Fund, a private fund focusing on suicide prevention.

## Conflict of Interest

MS has received support for one article in an MDEdge Child Psychiatry Consultation column on suicide prevention in primary care. CL has received consulting fees from Aetna Inc., CVS Health, and Lyra Health and HH has received research funding from Humana Inc. The remaining author declares that the research was conducted in the absence of any commercial or financial relationships that could be construed as a potential conflict of interest.

## Publisher's Note

All claims expressed in this article are solely those of the authors and do not necessarily represent those of their affiliated organizations, or those of the publisher, the editors and the reviewers. Any product that may be evaluated in this article, or claim that may be made by its manufacturer, is not guaranteed or endorsed by the publisher.

## Appendix

**Appendix 1:** Select tools for primary care practices.

**Resource**	**Focus**	**Website**	**Funding**
Suicide Prevention Resource Center	Policy and practice Toolkit for Primary Care Practices: Modules and practical resources for (1) getting started; (2) educating clinicians and office staff; (3) developing mental health partnerships; (4) patient management tools; (5) state resources, policy, and reimbursement; (6) health provider self-care; (7) patient education tools/resources	SPRC.org/settings/ primary-care	Housed at the University of Oklahoma Health Sciences Center, grants from the U.S. Department of Health and Human Services (HHS), Substance Abuse and Mental Health Services Administration (SAMHSA), Center for Mental Health Services (CMHS), under Grant No. 1H79SM083028-01
Zero Suicide	System wide organizational framework for safer suicide care Toolkit for systems leaders: lead, train, identify, engage, treat, transition, improve Resources particularly for integrated primary care settings	https://zerosuicide.edc.org/resources/settings/integrated-primary-care-and-behavioral-health	Education Development Center, the Suicide Prevention Resource Center, and the National Action Alliance for Suicide Prevention Funded by Universal Health Services (UHS), the Zero Suicide Institute at EDC, and the Substance Abuse and Mental Health Services Administration (SAMHSA), U.S. Department of Health and Human Services (DHHS) (grant 1 U79 SM0559945)
Ask Suicide Screening Questions (ASQ)	Screening and treatment flow diagrams for outpatients ages 10–24 Approved by the Joint Commission for use in all ages	https://www.nimh.nih.gov/research/research-conducted-at-nimh/asq-toolkit-materials	NIMH
The Lighthouse Project (formerly Center for Suicide Risk Assessment)	Columbia-Suicide Severity Rating Scale (C-SSRS) Screening with triage points for primary care settings Patients age 12+ C-SSRS also has an assessment tool that can be used by integrated mental health providers	https://cssrs.columbia.edu/the-columbia-scale-c-ssrs/healthcare/	The Research Foundation for Mental Hygiene Inc. Not for profit organization founded to help the New York State Department Office of Mental Health
Counseling on Access to Lethal Means (CALM Course)	Reducing access to lethal means like firearms and medication Free online course to (1) identify people who could benefit (2) ask about their access to lethal means (3) work with people and families to access mental health professionals and health care providers	https://www.sprc.org/resources-programs/calm-counseling-access-lethal-means	2015–2020 Suicide Prevention Resource Center Grant No. 5U79SM062297, awarded to EDC by the U.S. Department of Health and Human Services (HHS), Substance Abuse and Mental Health Services Administration (SAMHSA), Center for Mental Health Services (CMHS)
AIMS Center Protocols for Suicide Prevention in Primary Care	Protocol and workflow for suicidality response in clinics Empower staff to know what to do Keep patients/staff safe Follow up includes regularly tracking patients with risk, following up, and reviewing safety plans in the EMR	https://aims.uw.edu/resource-library/suicide-prevention-protocol	University of Washington Psychiatry and Behavioral Sciences

**Appendix 2:** Recommended reading and resources.

**Primary care clinicians**	**Primary care clinic administrators**
McDowell 2011 Brodsky 2018 Richards 2019 Norris 2021 Mann 2021	AIMS center: Developing Protocols for Suicide Prevention in Primary care Henry Ford Zero Suicide Prevention Guidelines Dueweke 2018 NAASP 2018 Turner 2020 Mann 2021
*Letter of introduction from PCP offices to local mental health colleagues:*	
https://www.sprc.org/sites/default/files/Section%203%20Developing%20Mental%20Health%20Partnerships.pdf	
*Caring Contacts examples:*	
https://nowmattersnow.org/wp-content/uploads/2020/04/Caring-Contacts-Text-and-Scripts.pdf	
*Patient safety plan templates:*	
https://suicidepreventionlifeline.org/wp-content/uploads/2016/08/Brown_StanleySafetyPlanTemplate.pdf	
*CALM training course:*	
https://zerosuicidetraining.edc.org/enrol/index.php?id=20	
*Billing:*	
PCP codes	
https://zerosuicide.edc.org/sites/default/files/Suicide%20Care%20Pathway%20Coding%20for%20Primary%20Care.pdf	
PCP/BH codes	
https://zerosuicide.edc.org/sites/default/files/Suicide%20Care%20Pathway%20Coding%20for%20Primary%20and%20Behavioral%20Health%20Care.pdf	
Integrated care codes	
https://www.cms.gov/Outreach-and-Education/Medicare-Learning-Network-MLN/MLNProducts/Downloads/BehavioralHealthIntegration.pdf	
